# Tibial Plateau Leveling Following Tibial Tuberosity Advancement: A Case Series

**DOI:** 10.3390/vetsci9010016

**Published:** 2022-01-01

**Authors:** Daniele Serrani, Pierre Paul Picavet, Juan Marti, Bernard Bouvy, Marc Balligand, Philip George Witte

**Affiliations:** 1 Southern Counties Veterinary Specialists, Forest Corner Farm, Hangersley, Ringwood BH24 3JW, UK; juan.marti@scvetspecialists.co.uk; 2 Department of Clinical Sciences, FARAH, Faculty of Veterinary Medicine, University of Liège, 4000 Liège, Belgium; pierre.picavet@uliege.be (P.P.P.); bbouvy@uliege.be (B.B.); marc.balligand@uliege.be (M.B.); 3 A30 Referrals, Station Rd, St Columb Major, Saint Columb TR9 6BX, UK; p.witte@penmellyn.co.uk

**Keywords:** TPLO, TTA, MMP, stifle instability, cranial cruciate ligament disease

## Abstract

Persistent stifle instability is a recognized complication following tibial tuberosity advancement techniques (TTAT). The aim of this study is to report the feasibility and outcome of tibial plateau leveling techniques (TPLT) to treat dogs with persistent lameness, suspected to be secondary to persistent stifle instability, following (TTAT). Medical records of dogs presented for persistent lameness after TTAT were reviewed. Preoperative data included orthopedic examination, lameness score and radiographs. Inclusion criteria included performance of a surgery to address persistent lameness and suspected instability. Short-term follow up data included orthopedic examination and radiographs of the stifle. Long-term follow up was based on postoperative Liverpool Osteoarthritis in Dogs (LOAD) questionnaire. Seven dogs were included in the study. Mean subjective preoperative lameness score was 3 ± 1.53. Mean preoperative patellar ligament angle relative to the tibial plateau (PLA^TP^) was 94° and mean tibial plateau angle (TPA) was 28°. Six dogs had tibial plateau leveling osteotomy and one had modified cranial closing wedge ostectomy. Mean postoperative PLA^TP^ was 79° and mean TPA was 5°. Mean subjective lameness score at follow up was 0.57 ± 0.49. Minor complications were present in 2 dogs and major complication in 1 dog. Mean LOAD questionnaire score was 6.6/52. TPLT can be performed after TTAT and may improve clinical function and stability in these cases in which persistent instability is suspected.

## 1. Introduction

Cranial cruciate ligament disease (CCLD) is the most common orthopaedic pathology affecting the hind limb of the dog [1]. According to two biomechanical theories, several techniques have been proposed to abolish cranial tibial thrust (CTT) associated with deficiency of the cranial cruciate ligament (CCL), either modifying the tibial plateau angle (TPA) or the patellar ligament angle (PLA) [2,3,4,5,6,7,8,9]. Between the formers, tibial plateau levelling osteotomy (TPLO) and cranial closing wedge ostectomy (CCWO) have been largely investigated. These techniques will be defined in this paper as tibial plateau levelling techniques (TPLT). The latter will be defined as tibial tuberosity advancement techniques (TTAT). Although few studies have compared the long-term outcome following these procedures, both TPLT and TTAT have been reported to be associated with excellent long-term outcomes [10,11,12]. Persistent stifle instability has been recognized as a possible postoperative complication after tibial tuberosity advancement (TTA) and TPLO [13,14]. To the authors’ knowledge, the use of TPLT is a hitherto unreported technique to treat persistent lameness and instability after TTAT. The primary aim of this retrospective study is to report the feasibility of TPLT to treat persistent lameness, thought to be associated with persistent cranio-caudal stifle instability after TTAT. The secondary aim is to provide short-term clinical outcome and long-term functional outcome of such surgeries.

## 2. Materials and Methods

Seven dogs with persistent lameness after TTAT, were presented at two referral centers (Southern Counties Veterinary Specialists, UK and Clinique Vétérinaire Universitaire of Liège University, BE) and treated with TPLT between July 2013 and October 2019. Orthopaedic examination was performed by three board certified surgeons prior to surgery and at least eight weeks following surgery. The lameness was subjectively graded from 0 to 5. The three board certified surgeons performed the surgeries. TTAT implants were partially or completely removed if required. Additional implants were used in combination with a TPLO plate, where considered necessary. Short term follow-up included a complete clinical and orthopaedic exam, and orthogonal radiographs of the affected hind limb. 

Case records were reviewed retrospectively and signalment, orthopaedic examination, perioperative radiographs and follow-up data were recorded. Pre and postoperative orthogonal radiographs were collected, and measurements made to determine the stifle flexion angle (SFA), the patellar ligament angle relative to the tibial plateau (PLA^TP^) and the TPA. Measurements were performed three times at one-week intervals by a surgery resident (D.S), and the mean was recorded. SFA, PLA^TP^ and TPA measurement was based on previously described methods [15]. Postoperative radiographs were evaluated for any evidence of implant failure, radiographic signs of infection and for bone healing. Intraoperative and postopearative complications were recorded. Postoperative complications were defined as minor, where resolution was achieved without surgical intervention, and major, where resolution was achieved with surgical intervention [16] Long term follow-up was based on email acquired Liverpool Osteoarthritis in Dogs questionnaire. 

## 3. Results

Seven dogs were included in the study: two Labrador retrievers, two mixed breeds, one Golden retriever, one English Springer spaniel and one West Highland white terrier, with a mean ± SD age and weight, respectively, of 84 ± 19.32 months and 25.69 ± 9.6 kg. 

TTAT were performed between 3 and 36 months prior to referral. None of the patients had ever regained complete limb function following the index TTA procedure. Cases 4, 6 and 7 had additional complications following the index surgery. Case 4 developed Organ/Space Surgical Site Infection and implant loosening two months following index surgery, requiring partial implant removal and antibiotic treatment prior to referral. Case 6 had previous implant associated complications, requiring partial-implant removal. Case 7 had revision surgery one year following index surgery for medial meniscal tear and deterioration of lameness approximately 36 months after the index surgery.

Mean ± SD subjective lameness score prior to surgery was 3 ± 1.53/5 (Table 1). 

Positive tibial compression test (TCT) was present in all the patients. Mean preoperative radiographic measurements were: SFA 112° (range: 104–137°), PLA^TP^ 94° (range: 83–102°) and TPA 28° (range: 22–34°). All the pre-operative radiographs showed signs of stifle effusion, and osteophytosis consistent with osteoarthritis (OA) associated with cranial cruciate ligament disease. Distal tibial crest fractures were present in Cases 1, 4 and 5. Radiographs of Case 4 showed radiolucency surrounding the implants compatible with failure of osteo-integration. Radiographs of Case 6 showed failure of the two staples components. 

A medial mini parapatellar arthrotomy was performed in Cases 1, 2, 3 and 7; the menisci were examined by visualization and probing. Cases 1 and 3 had intact menisci. Case 2 had a large bucket handle tear of the caudal horn of the medial meniscus and was treated with a partial meniscectomy. Case 7, which had already undergone a revision surgery for a meniscal tear of the caudal horn of the medial meniscus, had a severe tear of the cranial horn of the medial meniscus. Sub-total meniscectomy was performed. In Cases 4, 5 and 6 the stifle joint was not inspected because of surgeon preference.

Index TTAT and revision TPLT are reported in Table 2.

**Figure 1:**
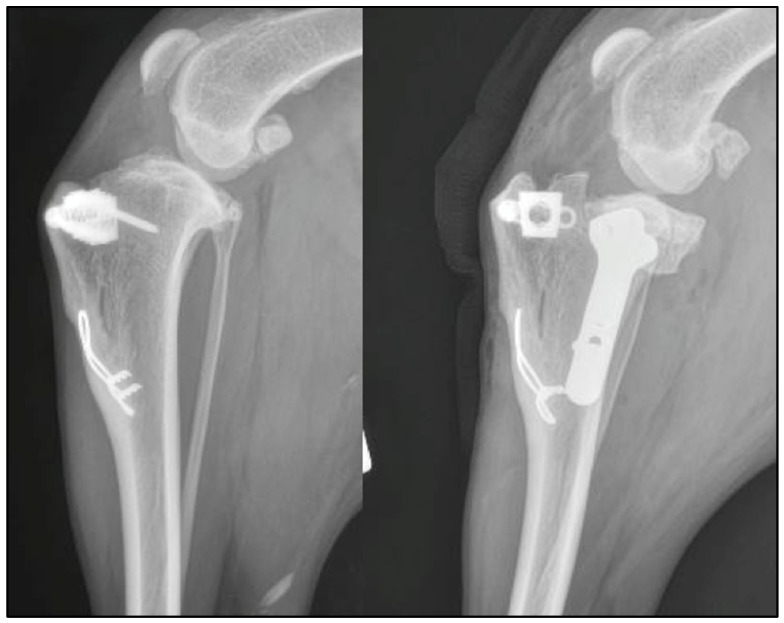
Case 7: pre- and post-operative mediolateral radiographs of the stifle. The caudal screw of the MMT cage was removed and a TPLO performed caudal to the implant.

In Case 4 the MMP implant was removed and submitted for culture and sensitivity testing, revealing no bacterial growth. In Case 5 the MMP pin and tension band components were removed to perform the radial osteotomy of the TPLO and were replaced following application of the TPLO plate. In Case 6, the failed staples and the K-wire components of the MMP were removed before performing the TPLO. In Case 7, the caudal screw of the TTA cage was removed to avoid the risk of interaction with the radial cut of the TPLO (Figure 1). 

All surgeries were successfully completed, and no intraoperative complications were experienced. Mean postoperative radiographic SFA, PLA^TP^ and TPA were, respectively, 115° (range: 105–129°), 79° (range: 77–87°) and 5° (range: 0–9°). 

Minor post-operative complications were recorded in Cases 3 and 6. Case 3 had radiographic evidence of patellar ligament thickening at the 8-week follow-up. However, due to the satisfactory evolution of the clinical function, no further investigation was sought. Case 6 had a recurrent episode of lameness approximately 8 weeks following the surgical procedure. Modulation of exercise was reintroduced and at the 12 weeks follow up, no lameness was detected. One major postoperative complication occurred in Case 4. Despite improvement in clinical function and adequate bone healing, the TPLO and K-wire implants were removed at the 8-week follow up for suspected soft tissues impingement associated with the wires.

Mean short term follow up ± SD was 10.8 ± 4.1 weeks. Mean subjective lameness score at the follow up was 0.57 ± 0.49. TCT was negative in all the patients. All tibial crest fractures demonstrated radiographic healing. 

LOAD questionnaire was available for 6/7 cases. Mean post-surgical time was 38 months (range 8–83 months) and no patients were receiving medication at the time of the questionnaire. Mean results of LOAD questionnaire was 6.7/52 (range 2–20). Clinical, radiographic findings and LOAD questionnaire results are reported in Table 3.

## 4. Discussion

**Figure 2:**
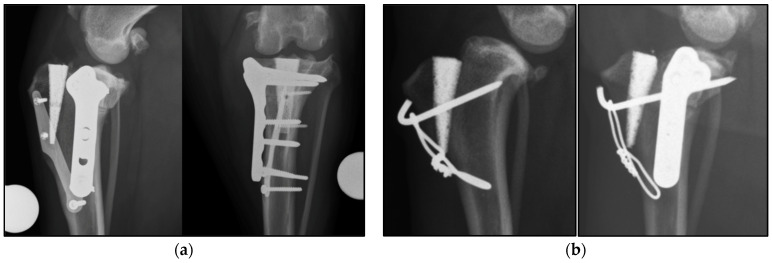
(**a**) 8 weeks postoperative mediolateral and craniocaudal radiographs of Case 1. The proximal tibial osteotomy was performed caudal to the TTAT implants. No interaction between the TPLO plate and the index implants occurred. (**b**) Preoperative and 8 weeks postoperative mediolateral radiographs of Case 5. The tension band was removed before performing the TPLO to avoid interaction of the pin with the osteotomy and replaced at the end of the procedure.

To the best of our knowledge, this is the first study documenting the feasibility of TPLT to treat persistent lameness and suspected instability following TTAT. TPLT were all successfully completed with or without removal of the TTAT implants (Figure 2) and none of the cases developed intraoperative complications. 

In Cases 4 and 6, the original implants were removed for reasons not associated with the surgical procedures, while in Cases 5 and 7 the implants were partially removed to prevent possible interactions with the cylindrical TPLO blade. All the patients were persistently lame following the index surgery and had persistent positive TCT. All patients showed decreased lameness score, subjectively assessed stifle stability (negative TCT), and improvement in clinical function at short term follow up. All dogs had a postoperative reduction of the estimated PLA^TP^ and TPA. Based on validated owner questionnaire progression of OA was graded as mild in 5/6 cases and moderate in 1/6. 

Persistent stifle instability is a possible complication following proximal tibial osteotomies in dogs. The reported rate of persistent instability seems to be higher following TTAT, in comparison to TPLT [13,14]. A direct correlation between persistent instability and suboptimal limb function has not been established, yet. However, persistent instability can lead to progression of osteoarthritis, late meniscal tears and has been theorized to contribute to “pivot shift phenomenon’’ [3,17,18]. The higher rate of stifle instability after TTA has been proposed as a possible explanation for the higher incidence of late meniscal injuries reported in the literature, in comparison to TPLO [13,14].

**Figure 3:**
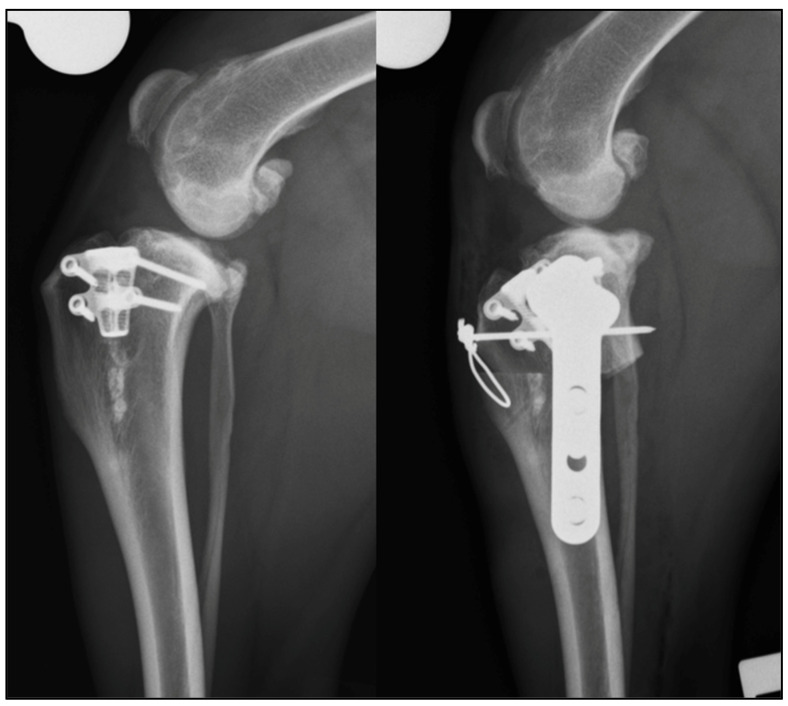
Case 2: medio-lateral pre- and post-operative radiographs of the left stifle. A modified CCWO has been performed and a TPLO plate positioned caudal to the TTA-rapid implant.

In this study 2/4 cases, in which arthrotomy was performed to inspect the menisci, had a meniscal injury. The concurrent partial meniscectomy complicates ascribing the clinical improvement in Case 2 to mCCWO alone (Figure 3). 

Interestingly, Case 7 had recurrent meniscal injuries, having already been treated by partial meniscectomy prior to referral. Although isolated meniscal tears have been reported in dogs [19,20], it is our suspicion that the persistent instability of the stifle played a primary role in the recurrence of meniscal lesions. As with Case 2, it is possible that the recurrent meniscal lesion in Case 7 was the main cause of lameness. 

Cases 4, 5 and 6 were not investigated for meniscal tears because of surgeon preference. In the veterinary literature there is still lack of evidence regarding the outcome of untreated meniscal injuries [21]. Since it has been anecdotally reported that clinical function following meniscal injury may improve in the absence of meniscal intervention, it is possible that the improvement in clinical function of these patients was secondary to spontaneous remission of a meniscal injury rather than to the TPLO performed. 

The cause of persistent instability after TTAT is still unknown but two proposed explanations include faults in the technical planning/execution of the procedure or in the biomechanical theory [22]. TTAT relies on the advancement of the tibial tuberosity such that the resulting force acting on the tibial tuberosity during the mid-stance phase of the gait, when the stifle is at 135°, becomes perpendicular to the tibial plateau [15]. Insufficient advancement of the tibial tuberosity may lead to a PLA > 90° at the mid stance phase of the gait and, theoretically, could be responsible for persistent cranio-caudal tibial subluxation [15]. 

In order to achieve an appropriate advancement of the tibial tuberosity that should provide stability during the gait, meticulous preoperative planning is mandatory. Several radiographic methods to estimate the SFA have been proposed and all of them can influence the final PLA [23]. However, to the author’s knowledge, none of these methods has been validated to be clinically superior for a correct preoperative planning. Moreover, there is a lack of studies in the veterinary literature comparing the variation of the SFA during the gait of different dog breeds, particularly for small breed dogs and chondrodystrophic breeds, and it is possible that the 135° angle may not be appropriate for TTAT planning in all the patients. A recent fluoroscopic kinematics study showed that mean SFA during the stance phase of dogs weighing between 20–40 kg ranged from 137–147° [24]. The SFA is important because it is linearly correlated to the PLA^TP^ [25]. The common tangent method (PLA^CT^) has been proposed as an alternative to the PLA^TP^ and seems to be less influenced by the SFA [25] and it has been suggested to be more biomechanically accurate than PLA^TP^ to determine the correct advancement of the tibial tuberosity required to achieve stability [15,25]. However, the PLA^TP^ method has been reported to have higher inter and intra operator accuracy [26].Another important step in the preoperative plan is the choice of the correct cage for the advancement of the tibial tuberosity. Several discrepancies between theoretical advancement required and the real in vivo advancement have been reported, depending on the technique used, the tibial conformation and the insertion point of the patellar ligament [27,28,29]. 

In this study, preoperative radiographs were obtained to plan the tibial plateau leveling procedures. The preoperative planning of TPLT does not require an SFA of 135° [15]. Even if only Case 5 had an estimated preoperative SFA of 137°, the mean preoperative SFA of all the other cases was significantly lower than 135° and only Case 3 and 7 had a preoperative PLA^TP^ of, respectively, 90° and 83°. For this reason, and considering that SFA and PLA are linearly correlated, it can be hypothesized that at least 5/7 TTAT were theoretically under-advanced before the surgical revision. As a result of the different SFA on the preoperative and postoperative radiographs, it is difficult to compare our perioperative PLA^TP^. However, all the postoperative PLA^TP^ were <90° and the mean PLA^TP^, even if evaluated at different SFA pre-and post-operatively, decreased by a mean 14.65°.

Under-advancement of the tibial tuberosity should lead to persistent cranial tibial translation during quadriceps contraction. For this reason, revision surgery might have been performed with the aim of further advancing the tibial tuberosity instead of leveling the tibial plateau. The surgeons chose TPLT for the revision of these procedures for two main reasons. First of all, due to the anatomy of the proximal tibia, TPLT can be performed without or with minimal implant removal while the osteotomy/ostectomy can be performed caudal (TPLO) or distal (CCWO) to TTAT implants and the TPLT plate can be positioned just caudal to them. This is an important preoperative consideration due to the fact that removing an osteo-integrated TTAT implant is a challenge and increases the surgical time and the risk of iatrogenic fracture of the tibial crest. Secondly, reducing the tibial plateau slope with TPLT reduces the PLA^TP^. The final mean postoperative TPA in this case series was 5°, and there is biomechanical evidence that reducing the TPA to 12° might be enough to bring the PLA^CT^ to 90° on the mid-stance phase of the gait [30]. 

The effect of the combination of TPLT and TTAT on the caudal cruciate ligament (CaCL) is unknown. Sumner et al. reported that the CaCL was damaged in the 88% and completely disrupted in the 25% of dogs enrolled in their study [31]. A human study demonstrated that TTA induces increased CaCL and tibiofemoral contact forces at larger flexion angles [32], and TPLO may induce increased strain on the CaCL as well, especially in case of over rotation of the tibial fragment [33]. In all our cases, the postoperative PLA^TP^, even if not estimated at 135°, was significantly lower than 90°, and the long-term effect of a low TPA combined with a low PLA is a possible concern. Considering that differentiation by clinical examination of cranial and caudal drawer sign can be challenging, inspection of the CaCL before performing the TPLO would be recommended. 

In Case 3 radiographic evidence of patellar ligament thickening (PLT) was apparent at the eight week follow up. Postoperative PLT is a common finding after TPLO and TTA [34]. PLT may be an incidental finding and may not be associated with a clinically relevant desmopathy; however, PLT after TPLO has been also described as possible cause of persistent lameness, delayed union of the fracture gap and prolonged recovery [35]. Due to the fact that patellar desmopathy can be a self-limiting condition, and that the overall clinical function of the dog was considered satisfactory for the postoperative period, the patellar ligament thickening was not investigated. A risk factor for PLT has been reported to be a final TPA < 6° [35,36]. Case 3 had a final TPA of 4° and it is possible that this contributed to the development of PLT, however the effects of a combined tibial plateau leveling with a TTA on the patellar ligament has not been investigated yet. Theoretically, advancement of the tibial tuberosity increases the moment arm of the patellar ligament mechanism and consequently reduces the torque required to extend the stifle. Leveling of the tibial plateau reduces the moment arm of this mechanism and therefore increases the torque required to extend the stifle [15]. For this reason, it has been theorized that TPLO should cause more patellar desmopathy than TTA [15]. However, the long-term effect of the combination of these procedures on the patellar ligament is unknown. 

Case 4 developed a surgical site infection following the index surgery and had a surgical implant revision prior to referral. The absence of osteo-integration of the implant, was considered the most likely cause of the persistent lameness. Removing the implant and proceeding with the TPLO, without treating the infection first, was strongly discouraged but, unfortunately, requested by the owner due to financial limitations. 

Cases 1, 4 and 5 had concurrent tibial crest fractures. It is not clear if tibial crest fracture may have played a role in the clinical deterioration of the patients or not. However, all the fractures were healing at the 8 weeks follow up. 

The resulting of the LOAD questionnaire for Case 2 was significantly higher than the other Cases (score of 20/52), however, the owner of this patient reported that the dog was lame on the contralateral hind limb but choose not to investigate or treat the lameness. Contralateral hind limb lameness in this case may have influenced the results of the LOAD questionnaire. 

This study has several limitations including its retrospective nature, the limited number of patients, the absence of complete data for all of them and the absence of an objective long term outcome measurement. Persistent cranio-caudal instability following TTAT is difficult to confirm clinically because TTAT relies on the active contraction of the quadriceps muscle during the gait [5,37] and no test is validated to clinically evaluate in vivo stifle stability after TTAT [13]. 

Accuracy of radiographic measurements in this study was limited by variable radiographic limb positioning. The absence of the entire femur to define the SFA complicated objective confirmation of under-advancement of the tibial tuberosity. A kinetic and kinematic evaluation of the patients before and after the surgery may have objectively confirmed our pre- and post-operative subjective evaluation of the lameness. Postoperative loaded horizontal radiographs, or a dynamic fluoroscopic evaluation of the patient at walk and trot would have provided more objective data to confirm the persistent functional stifle instability. Case 7 died of cause not related to the surgery and the long term follow up was missing. We decided to include this dog in the series to illustrate the feasibility of TPLT following a larger variety of TTAT. Finally, long term follow up was only based on a validated owner questionnaire for the evaluation of OA in dogs. It is possible that the results of the questionnaires were biased by concomitant orthopaedic conditions. 

## 5. Conclusions

In conclusion, TPLT were successfully completed after TTAT to treat persistent lameness, with or without partial or complete implant removal. TPLT may provide good short-term outcome and improvement of the clinical function and mild long-term progression of signs of osteoarthritis. Clinicians faced with persistent lameness, suspected to be associated to persistent stifle instability, following TTAT, for example in those cases in which adequate advancement of the tibial tuberosity is not achieved, may consider TPLT to improve stifle function and stability.

## Figures and Tables

**Table 1:** Clinical data.

Case	Breed	Age	Sex	Weight	Previous Surgery	Deterioration/Onset of Lameness	Hindlimb Lameness
1	Golden retriever	60 months	M	40.0	3 months	Lame since index surgery and deterioration in the last 4 weeks	2/5 right
2	Mongrel	84 months	F	26.3	36 months	Lame since index surgery and deterioration in the last 6 months	3/5 left
3	Springer Spaniel	72 months	M	19.4	8 months	Lame since index surgery	2/5 right
4	Labrador Retriever	88 months	M	31.5	3 months	Lame since index surgery	2/5 right
5	West Highland White Terrier	92 months	F	10.85	3 months	Lame since index surgery and deterioration in the last 6 weeks	5/5 right
6	Labrador Retriever	72 months	M	31.3	36 months	Lame since index surgery and deterioration in the last 6 months	3/5 right
7	Mongrel	120 months	F	20.5	36 months	Lame since index surgery and deterioration in the last week	5/5 right

**Table 2:** Index surgeries, preoperative measurements, revision surgeries and postoperative measurements.

Case	TTAT	Preop SFA	Preop PLA^TP^	Preop TPA	TPLT	Stifle Inspection	Postop SFA	Postop PTA^TP^	Postop TPA
1	Modified Maquet tibial tuberosity advancement	104°	94 °	28°	TPLO	Complete CCL rupture, intact menisci	109°	77°	9°
2	TTA rapid	112°	96°	32°	Modified CCWO (mCCWO)	Complete CCL rupture and medial meniscal tear (caudal horn)	129°	87°	0°
3	Modified Maquet Procedure (MMP)	104°	90°	24°	TPLO	Partial CCL rupture and intact menisci	105°	74°	4°
4	MMP	108°	96°	26°	TPLO and tension band	N/A	106°	86°	9°
5	MMP	137°	102°	34°	TPLO and tension band	N/A	117 °	79°	4°
6	MMP with 2 staples	111°	95°	27°	TPLO	N/A	115°	79°	8°
7	Modified Maquet Technique (MMT)	108°	83°	22°	TPLO	Complete CCL rupture and medial meniscal tear (cranial horn)	127°	70°	1°

**Table 3:** Clinical, radiographic findings and LOAD questionnaire results.

Case	Short-Term Follow Up	Subjective Lameness Score	Clinical Findings	Radiographic Findings	Long Term Follow Up	LOAD Score
1	8 weeks	0	Mild muscle atrophy, mild reduction of range of motion (ROM), negative TCT	Good healing of the bone, tibial tuberosity fracture healed and moderate stifle effusion	61 months	9/52
2	8 weeks	1	Very mild muscle atrophy, mild reduction of ROM, negative TCT	N/A	17 months	20/52
3	8 weeks	1	Mild muscle atrophy, mild reduction of the ROM, negative TCT	Almost complete bone healing, absence of implant associated complication, patellar ligament thickening	42 months	2/52
4	12 weeks	1	Moderate muscle atrophy, mild reduction of ROM, negative TCT	Good healing of the bone, healing of the tibial tuberosity fracture, mild stifle effusion	83 months	3/52
5	8 weeks	1	Mild muscle atrophy, normal ROM, negative TCT	Almost complete healing of the bone and no signs of stifle effusion	8 months	4/32
6	12 weeks	0	No muscle atrophy, mild reduction in ROM, negative TCT	Complete healing of the bone and mild stifle effusion	17 months	2/52
7	20 weeks	0	No muscle atrophy, negative TCT	N/A	N/A (dog died of kidney disease)	N/A

## Data Availability

The Data presented in this study are available within the article.
